# Tumour necrosis factor-α (TNF-α) enhances dietary carcinogen-induced DNA damage in colorectal cancer epithelial cells through activation of JNK signaling pathway

**DOI:** 10.1016/j.tox.2021.152806

**Published:** 2021-06-15

**Authors:** Aminah G. Alotaibi, Jia V. Li, Nigel J. Gooderham

**Affiliations:** a Section of Biomolecular Medicine; b Section of Nutrition Research, Division of Digestive Diseases, Department of Metabolism, Digestion and Reproduction, Faculty of Medicine, Imperial College London, London, UK; c National Centre for Genomic Technology, King Abdulaziz City for Science and Technology, KACST, Riyadh, Saudi Arabia

**Keywords:** TNF-**α**, Genotoxicity, Cytotoxicity, DNA damage, JNK pathway, CYP1A1/1B1

## Abstract

Colorectal cancer (CRC) is the third most common cancer worldwide and the second leading cause of cancer death. Benzo[a]pyrene (BaP) and 2-amino-1-methyl-6-phenylimidazol [4,5-b] pyridine (PhIP) present in cooked meat are pro-carcinogens and considered to be potential risk factors for CRC. Their carcinogenic and mutagenic effects require metabolic activation primarily by cytochrome P450 1 family enzymes (CYPs); the expression of these enzymes can be modulated by aryl hydrocarbon receptor (AhR) activation and the tumour microenvironment, involving mediators of inflammation. In this study, we hypothesized that tumour necrosis factor-α (TNF-α), a key mediator of inflammation, modulates BaP- and PhIP-induced DNA damage in colon cancer epithelial cells. Importantly, we observed that TNF-α alone (0.1–100 pg/ml) induced DNA damage (micronuclei formation) in HCT-116 cells and co-treatment of TNF-α with BaP or PhIP showed higher levels of DNA damage compared to the individual single treatments. TNF-α alone or in combination with BaP or PhIP did not affect the expression levels of CYP1A1 and CYP1B1 (target genes of AhR signaling pathways). The DNA damage induced by TNF-α was elevated in p53 null HTC-116 cells compared to wild type cells, suggesting that TNF-α-induced DNA damage is suppressed by functional p53. In contrast, p53 status failed to affect BaP and PhIP induced micronucleus frequency. Furthermore, JNK and NF-κB signaling pathway were activated by TNF-α treatment but only inhibition of JNK significantly reduced TNF-α-induced DNA damage. Collectively, these findings suggest that TNF-α induced DNA damage involves JNK signaling pathway rather than AhR and NF-κB pathways in colon cancer epithelial cells.

## 1 Introduction

Colorectal cancer (CRC) is the third most common cancer worldwide and the second leading cause of cancer death (Haggar and Boushey, 2009). The majority of CRC is sporadic and attributable to environmental factor-induced gene mutations and inflammation (Simon, 2016). Benzo(a)pyrene (BaP) and 2-amino-1 methyl-6-phenylimidazo [4,5-b] pyridine (PhIP), dietary carcinogens present in cooked meat, are potential risk factors contributing to the development of CRC (Gooderham et al., 1997; Cross and Sinha, 2004). BaP is a potent ligand of the aryl hydrocarbon receptor (AhR), the activation of which regulates cytochrome P450 1A1 (*CYP1A1*) and *CYP1B1* genes (Nebert and Dalton, 2006). In turn, the CYP1 family enzymes are required to catalyze BaP and PhIP into their genotoxic metabolites, 7,8-diol-9,10 epoxy BaP and *N*-hydroxy PhIP, respectively, to exert their mutagenic and carcinogenic effects (Lodovici et al., 2004; Zhao et al., 1994; Gooderham et al., 1997, 2002). These genotoxic metabolites covalently bind to DNA, particularly guanine bases and disrupt the double-helical structure, resulting in DNA damage and mutations (Lodovici et al., 2004; Sinha et al., 2005; Yadollahi-Farsani et al., 1996). The CYP 1 family of enzymes comprise three members, 1A1, 1A2 and 1B1. In humans, all three members are capable of metabolizing BaP and PhIP to their genotoxic derivatives. However, CYP1A2 is predominantly expressed hepatically and the other two family members are predominantly extra-hepatic (Zhao et al., 1994; Boobis et al., 1994; Crofts et al., 1998). CYP1A1/1B1 are major CYP enzymes responsible for the metabolic activation of many chemical carcinogens, and their high expression frequencies were reported in many cancers such as colon cancer, lung, brain and lymphomas (Nebert and Dalton, 2006).

Local inflammation conditions in the colon, such as colitis, is a strong risk factor in CRC development and progression (Balkwill et al., 2005). It is also known that inflammatory mediators in the tumour microenvironment are involved in crosstalk between immune and cancer cells (Patel and Gooderham, 2015a, 2015b). Tumor necrosis factor-α (TNF-α) is a pleiotropic proinflammatory cytokine, which is involved in pathophysiology of inflammatory bowel disease (IBD) (Schwabe and Brenner, 2006) and present at high levels in serum and tissues of CRC patients (Stanilov et al., 2014; Al Obeed et al., 2014). TNF-α has been shown to suppress cellular responses to AhR activation (Morgan, 2001) and has been considered as an anti-tumor cytokine owing to its strong cytotoxic effects on some tumor cells *in vitro* (Watanabe et al., 1988). Since TNF-α treatment induces high systemic toxicity, it can only be used as a tumor therapeutic agent under conditions where the systemic TNF-α action was reduced or prevented (Talmadge et al., 1988). Conversely, TNF-α has been shown to inhibit anti-tumor immune responses through modulating leukocytes and altering cancer cell phenotypes during cancer progression (Montfort et al., 2019).

TNF-α exerts its effects by binding to TNF-α receptors, TNFR1 and TNFR2, which can activate signaling pathways including c-Jun N-terminal kinase (JNK), nuclear factor-kappa B (NF-κB) and a caspase cascade in human cancer cells (Zhang et al., 2004). Activation of the tumor suppressor p53 by damage and stress stimuli often correlates with induction of JNK (Montfort et al., 2019). The tumor suppressor protein p53 is a master regulator of the genome and via p21 can arrest cell cycle upon DNA damage, activate DNA repair proteins or initiate apoptosis if DNA damage is irreparable; therefore, p53 is crucial for genomic stability (Mager et al., 2016). Furthermore, it has been reported that TNF-α suppressed the expression of CYP1A1 in human primary hepatocytes, likely through NF-κB activation, since a mutual inhibitory crosstalk between the NF-κB and AhR signaling pathway has been demonstrated in hepatic cells (Tian et al., 2002). Yet paradoxically TNF-α has been shown to enhance the genotoxic effect of BaP in hepatic cells *via* upregulation of CYP1B1 (Umannová et al., 2008a) Which of these TNF-α mediated outcomes applies in colorectal cells is not clear, but it seems likely that either outcome will impact on the genotoxicity of dietary carcinogens such as BaP and PhIP in these cells.

## 2 Materials and methods

### 2.1 Cell culture

The human colorectal adenocarcinoma cell line HCT116p53+/+ (WT) were obtained from ATCC (LGC Prochem, Middlesex, UK). HCT116p53-/- Cells were purchased from Horizon Discovery, Cambridge, UK. Cells were cultured in RPM1640 medium supplemented with 10 % Fetal Bovine Serum (FBS), antibiotics penicillin (100 units/mL) and streptomycin (100 μg/mL), and 2 mM L-glutamine (GIBCO, Life technologies). Cells were incubated at 37 °C in a humidified atmosphere with 5% CO_2_. Cells at passages between 3–8 were used for the study.

### 2.2 Cell treatment

All the cells were maintained in RPM1640 medium supplemented with 5% dextran charcoal stripped FBS for at least 72 h prior to treatment. Cells were cultured at a density of 1 × 10^5^ cells per well in a 24-well plate. Recombinant human TNF-α (Merck, Sigma-Aldrich, Darmstadt, Germany) was dissolved in phosphate buffer saline (PBS) containing 0.1 % human serum albumin (Sigma-Aldrich) and then added to cell cultures at the following doses, 0.01, 0.1, 1, 10, 100 pg/mL for varying times of incubation (i.e. 24, 48 and 72 h); physiological concentration of TNF-α has been reported to be ∼10 pg/mL (Wyble et al., 1996). BaP and PhIP, obtained from Sigma-Aldrich and Toronto Research Chemical Inc. (Toronto, Canada), respectively, were prepared in dimethyl sulfoxide (DMSO). The treatment of cells with BaP was at 0.1, 1, 10 μM, and PhIP was at 1, 10, 100 μM. An NF-kB inhibitor, Bortezomib (BZ) (LC laboratories), was used at 0.13 μM 4.5 h prior to TNF-α treatment. A JNK inhibitor, SP600125 (Abcam, UK), was used at 30 μM and co-treated with TNF-α. Both inhibitors were dissolved in DMSO. The concentration of DMSO in all incubates was kept constant at 0.5 %.

### 2.3 Cytotoxicity and genotoxicity

Cytotoxicity was determined by counting cells using Moxi Z automated cell counter (ORFLO Technologies, USA). The Micronucleus (MN) assay is commonly used to detect genotoxic potential (clastogenic and aneugenic) of carcinogens in many cell types. MN assay was performed in line with OECD guidance, adapted for HCT116 cells, but in the absence of a mitotic inhibitor (Patel and Gooderham, 2015b). Cells were cultured at a density of 4 × 10^4^ cells per well in a 24-well plate and treated with TNF-α, BaP or PhIP in an optimized protocol as detailed previously (Patel and Gooderham, 2015b). Etoposide (Sigma-Aldrich) was used as a positive control at a concentration of 125 nM. Cells were harvested and resuspended in culture medium containing 2% pluronic (GIBCO, Life technologies), then deposited as monolayer on microscope slides at density 3 × 10^4^ per slide by Cytospin Centrifugation (Thermo Scientific) at 100 g for 5 min. Cells were dried at room temperature for 2 h. Cells were fixed with 100 % methanol and stained for 60 s with acridine orange 0.1 mg/mL dissolved in PBS (Sigma Aldrich). MN frequencies were scored blind for at least 2000 cells per data point under a microscope and three biological replicates were performed per treatment.

### 2.4 Reactive oxygen species (ROS)

Oxidative stress was determined by measuring the production of reactive oxygen species (ROS). Diclorofluorescein (DCF) method was used to determine ROS production fluorometrically. HCT116 cells (2 × 10^4^ /well) were seeded into 24 well plates in 10 % FBS overnight then replaced with1% FBS media (1 mL). Carboxy-H2DCFDA (20 μL of 30μM, oxidative stress indicator) (Invitrogen, United Kingdom) was added to cells and incubated for 30 min. at 37 °C then washed with PBS and new media (1% FBS). Cells were then treated with TNF (0.01–100 pg/ml) and incubated over 48 h. Fluorescent measurements (excitation at 485 nm and emission at 520 nm) were measured using a dynamic assay from 10 min. −48 h on florescent plate reader (BMG POLARstar Galaxy Labtech, Ortenberg, Germany). The intensity of fluorescence is proportional to intracellular ROS levels. Under these conditions, treatment of cells with the positive control (hydrogen peroxide) gave a sustained cumulative induction of fluorescence over the first 24 h of incubation and remained elevated over the 48 h of incubation, compared to the vehicle control.

### 2.5 RNA extraction

Total RNA was extracted from cells using RNeasy Mini Kit reagents (QIAGEN, Hilden, Germany) according to the manufacture’s protocols. RNA products were quantified by nano-spectrophotometer (Implen, Essex, UK). The RNA purity was assessed based on 260/280 nM and 260/230 nM ratios. The extracted RNA samples were stored at −80 °C until required.

### 2.6 Reverse transcription and qPCR

mRNA reverse transcription and qPCR was performed as previously described (Patel et al., 2014) using superscript II reverse transcription kit (Invitrogen, Life Technologies). qPCR was performed using predesigned expression assays for CYP1A1(Hs01054797_g1), CYP1B1 (Hs00164385_m1) and GAPDH(Hs99999905_m1) (TaqMan, Applied Biosystems, Life Technologies). Fast PCR master mix was used according to manufacturer’s protocol (TaqMan, Applied Biosystems, Life Technologies).

### 2.7 Immunoblotting

Cells were cultured in 60 mm dishes and the cell treatment was carried out as described above. Cells were scraped and lysed in cell lysis buffer (1X Cell Lysis Buffer: 20 mM Tris−HCl (pH 7.5), 150 mM NaCl, 1 mM Na_2_EDTA, 1 mM EGTA, 1% Triton, 2.5 mM sodium pyrophosphate, 1 mM β-glycerophosphate, 1 mM Na_3_VO_4_, 1 μg/mL leupeptin) obtained from Cell Signaling Technology Inc., UK, for 30 min on ice. The lysate was centrifuged at 10,000 g for 10 min at 4 °C. Protein concentrations were determined by Bio-Rad protein assay (Bio-Rad, laboratories Inc., UK) as manufacture protocol. An amount of 60 μg of total cellular protein sample was loaded into 4–12 % polyacrylamide gel (Life Technologies, Paisley, UK) and electrophoresed for 1.5 h. The protein bands were transferred onto a polyvinylidene difluoride (PVDF) membrane, (Invitrogen, Life Technology, USA). The membrane was blocked using 5% non-fat dried milk dissolved in TBS-Tween buffer (25 mM Tris−HCl pH 7.4, 0.15 M NaCl and 0.05 % Tween-20) for 1 h at room temperature. Membranes were washed with TBS-Tween buffer and incubated with specific primary antibodies against phosphorylated (p)-JNK (1:500 cat. no. sc-6254), JNK (1:500; cat. no.sc-7345), phosphorylated (p) -IkB (1:500; cat. no sc-8404) and IkB (1:500; cat. no sc-1643) and beta-actin (used as a loading control) all Santa Cruz Biotechnology, Inc., Dallas, TX, USA) at 4 °C overnight. The blots were then incubated with HRP-conjugated anti-mouse antibodies (1:1000; Santa Cruz Biotechnology, Inc) for 1 h at room temperature. Immunoreactive proteins were visualized using Luminol Reagent (Santa Cruz, Biotechnology, Texas, USA) and images were captured and analyzed using GeneSnap Version 7.1 (Syngene, Cambridge, UK) and the intensity of blot bands was quantified with ImageJ 2.0 (National Institutes of Health, Bethesda, Maryland, USA). The ratio of phosphorylated protein to total protein (IkB or JNK), respective to the vehicle control, was determined.

### 2.8 Statistical analysis

Data from at least three independent biological replicates are presented as a mean ± standard error of the mean (SEM). Significant differences (P < 0.05) were determined using one-way analysis of variance (ANOVA) followed by a Dunnet post test (Graphpad Prism 8, GraphPad software Inc., La Jolla, CA., USA).

## 3 Results

### 3.1 Cytotoxic and genotoxic effects of TNF-α and co-treatment of TNF-α and dietary carcinogens on HTC116 cell line

**Fig. 1:**
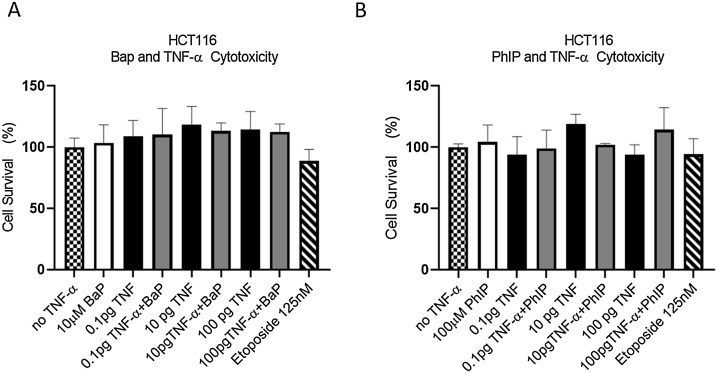
**Cytotoxicity of HCT116 cells. (A)** Cells treated with 10 μM BaP, TNF-α at doses of 0.1, 10, 100 pg/mL, or a combination of both for 48 h. (**B**) Cells treated with 100 μM PhIP, TNF-α at doses of 0.1, 10, 100 pg/mL, or a combination of both for 48 h. Cytotoxicity is expressed as % of viable cells relative to the negative control. Significance was calculated using one-way ANOVA with a Dunnett test. The data are presented as SEM from three independent cultures.

**Fig. 2:**
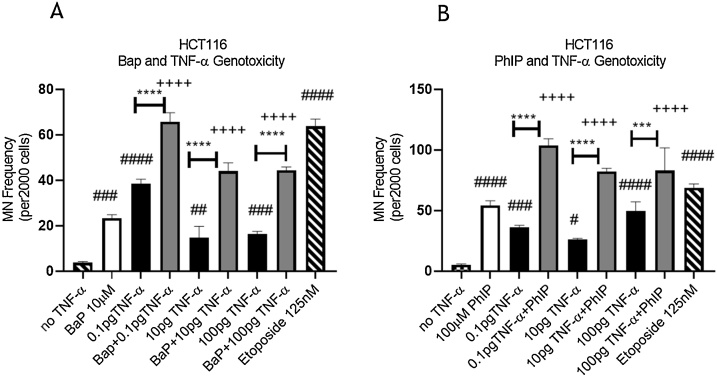
**Genotoxicity of HCT116 cells.** DNA damage measured by micronucleus frequency in **(A)** cells co-treated with TNF-α and BaP or **(B)** cells co-treated with TNF-α and PhIP for 48 h. The treatment concentrations of BaP and PhIP were 10 μM and 100 μM, respectively. Statistically significant differences are shown as following: carcinogen treatment vs. co-treatment (*p* < 0.05, ++*p* < 0.01, +++*p* < 0.001, ++++ *p* < 0.0001), TNF-α vs. co-treatment (*p < 0.05, **p < 0.01, ***p < 0.001, ****p < 0.0001), negative control Vs. carcinogens or TNF-α treatment (#*p* < 0.05, ##*p* < 0.01, ###*p* < 0.001, ####*p* < 0.0001). Significance was calculated using one-way ANOVA with a Dunnett test. The data are presented as SEM from three independent cultures.

At the concentrations used, cytotoxicity was not observed in cells treated with BaP, PhIP and TNF-α alone or in combination, compared to the respective vehicle control (Fig. 1). A micronucleus assay was used to determine the effect of cell treatments on genetic damage. Treatment with TNF-α (0.01–100 pg/ml) alone produced a strong positive response at the lowest dose employed (0.1 pg/mL) for the induction of micronuclei in HCT116 cells (Fig. 2A and B) compared to the vehicle control. The positive control etoposide gave a strong induced micronucleus response (Fig. 2A and B) in line with previous reports from our laboratory (Patel and Gooderham, 2015b; Malik et al., 2018; David et al., 2016; David and Gooderham, 2018).

BaP at 10 μM and PhIP at 100 μM induced a strong DNA damage response compared to the vehicle control (Fig. 2A and B). Interestingly, the co-treatment of TNF-α and the carcinogens induced a significantly higher MN frequency compared to the single individual treatments at all the tested concentrations with the most profound effect in combinations with the low dose of TNF-α (0.1 pg/mL) (Fig. 2A and B). These results demonstrated that co-treatment with TNF-α enhanced BaP- and PhIP-induced DNA damage in HCT116 cells.

### 3.2 TNF-α-induced DNA damage is not due to formation of Reactive Oxygen Species

In a dynamic assay with time points ranging from 5 min. to 48 h, TNF-α (0.01–100 pg/ml) failed to induce ROS in HCT116 cells compared to the negative vehicle control (data not shown); hydrogen peroxide positive control gave a sustained cumulative induction of ROS over the same period. These data suggest that TNF-a induced MN frequency in the HCT116 cells is not dependent on the generation of ROS.

### 3.3 TNF-α-induced DNA damage is independent of CYP1A1/B1 expression in HCT-116 wild type cells

**Fig. 3:**
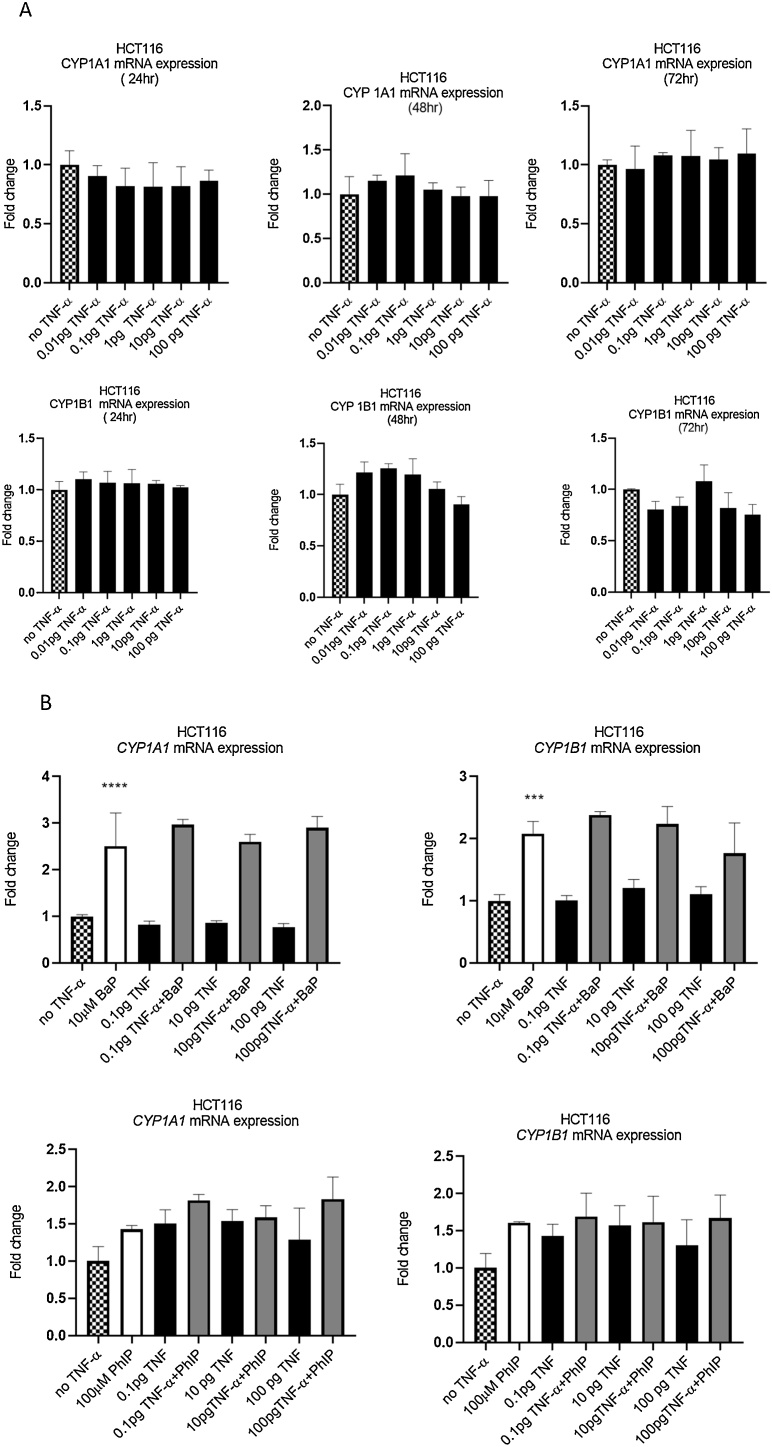
***CYP1A1/1B1* gene expression in HCT116**. **(A)** Cells were treated with TNF-α dose range of (0.01-100 pg/mL) over 72 h. **(B**) cells co- treated with BaP and PhIP. Gene expression were measured by RT-qPCR. Data are shown as fold change. Significance was calculated using one-way ANOVA with a Dunnett test (GraphPad Prism 8). The data are presented as SEM from three independent cultures.

The BaP and PhIP metabolic activation pathways require CYP1 family enzymes to produce their genotoxic metabolites (David et al., 2016); therefore, we investigated if TNF-α-induced genotoxicity and the enhanced genotoxicity by TNF-α in co-treatments are through the modulation of the extrahepatically expressed CYP1A1/1B1. As expected, expression of CYP1A1 and 1B1 mRNA was detected in HCT116 cells. Treatment of cells with BaP significantly induced the expression of CYP1A1 and CYP1B1 mRNA, whereas treatment with PhIP did not (Fig. 3B); this is consistent with the relative potencies of these two chemicals as ligands of the AhR for the induction of CYP1 family genes. TNF-α alone (Fig. 3A) or co-treatment with BaP or PhIP (Fig. 3B) did not significantly alter CYP1A1 and CYP1B1 mRNA expression over 72 h, indicating that TNF-α-induced DNA damage is not *via* induction of CYP1A1/1B1 expression.

### 3.4 TNF-α-induced DNA damage is dependent on p53 in HCT116 cells

**Fig. 4:**
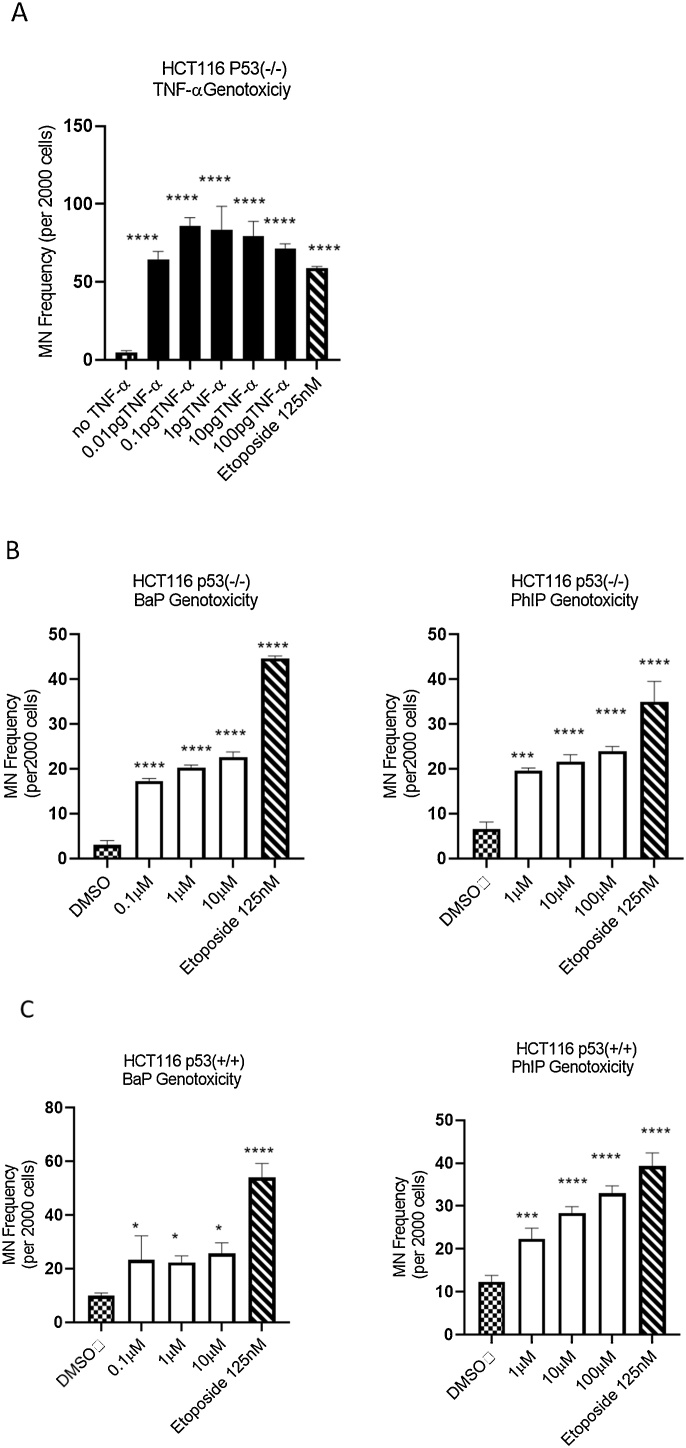
**Genotoxicity** in **HCT116 cells.** DNA damage measured by Micronucleus frequency in HCT116 p53-/- and HCT116 p53+/+ cells induced by **(A**) HCT116 p53-/- treated with TNF-α dose range of (0.01-100 pg/mL) and **(B**) HCT116 p53-/- treated with BaP dose range of (0.1, 1,10μM) and PhIP dose range of (1-100μM), (**C**) HCT116 p53+/+ treated with BaP and PhIP. Statistically Significant differences are shown for comparisons between treated samples Vs. vehicle (*p < 0.05, **p < 0.01, ***p < 0.001, **** *p* < 0.0001). Significance was calculated using one-way ANOVA with a Dunnett test (GraphPad Prism 8). The data are presented as SEM from three independent cultures.

p53 is a tumor suppressor protein and the activation of p53 in response to DNA damage promotes DNA repair (Zhu et al., 2005). We therefore considered the possibility that p53 may be involved in TNF-α-induced DNA damage. To examine this, we used HCT116 p53(-/-) cells. Significantly higher frequencies of micronucleus formation were observed in HCT116 p53(-/-) cells at all tested TNF-α concentrations compared to the corresponding vehicle control (Fig. 4A) and compared to the wild type HCT116 p53(+/+) (Fig. 2A & B). Whilst the treatments generated a non-linear dose-response that again peaked around 0.1 pg/mL in the HCT116 p53(-/-) cells (Fig. 4A), the magnitude of the response was greater (∼2 fold) compared with that observed in the HCT116 p53(+/+) wild type cells (Fig. 2A and B). In contrast, the MN frequencies of the cells treated with BaP, PhIP or etoposide (the positive control) were similar in both HCT116 p53(-/-) (Fig. 4B) and WT cells (Fig. 4C). These observations suggested that a functional p53 gene response attenuated TNF-α-induced genotoxicity, but not that of BaP, PhIP and etoposide.

### 3.5 JNK signaling pathway mediates TNF-α genotoxicity in HTC-116 wild type cells

To determine the mechanisms underlying TNF-α genotoxicity, we further investigated if signaling pathways of NF-kB and JNK are involved. JNK is phosphorylated by JNK- activating kinase (JNKK), members of the MEK family. Activation of NF-kB mainly occurs via IkB kinase-mediated phosphorylation. HTC-116 wild type cells were treated with TNF-α for 24 h and protein expression levels of IkB, phospho-IkB, JNK and phospho-JNK were measured using immunoblotting.

**Fig. 5:**
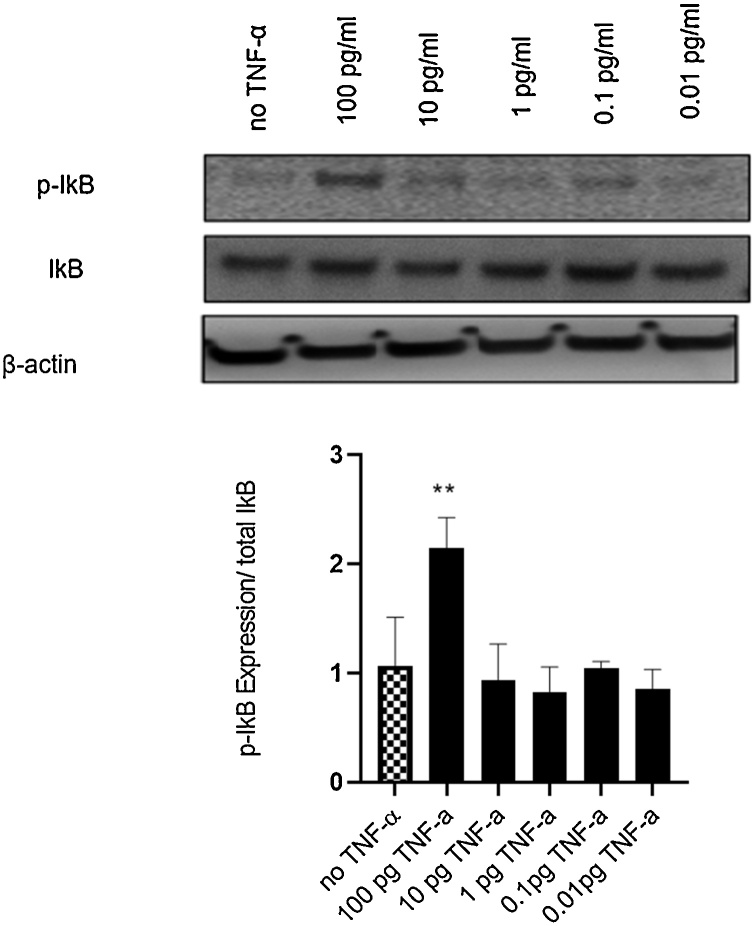
**Effect of TNF-α on NF-kB activation in colon cancer cells HCT116.** Cells were treated with TNF-α dose range of (0.01-100 pg/mL) for 24 h. IkB and phosphorylated IkB (p-IkB) protein expression were determined by immunoblotting and quantified using Image J. Beta-actin was a loading control. Significance was assessed using one-way ANOVA (GraphPad Prism 8, (*p < 0.05, **p < 0.01, ***p < 0.001, **** *p* < 0.0001) compared to control. Data are presented as the ratio of p-IkB/total IkB with SEM from three independent cultures(n=3).

TNF-α treatment increased the ratio of phospho-IkB to total IkB levels significantly but only at the highest concentration of 100 pg/mL, compared with vehicle control (Fig. 5), confirming activation of the NF-κB pathway.

**Fig. 6:**
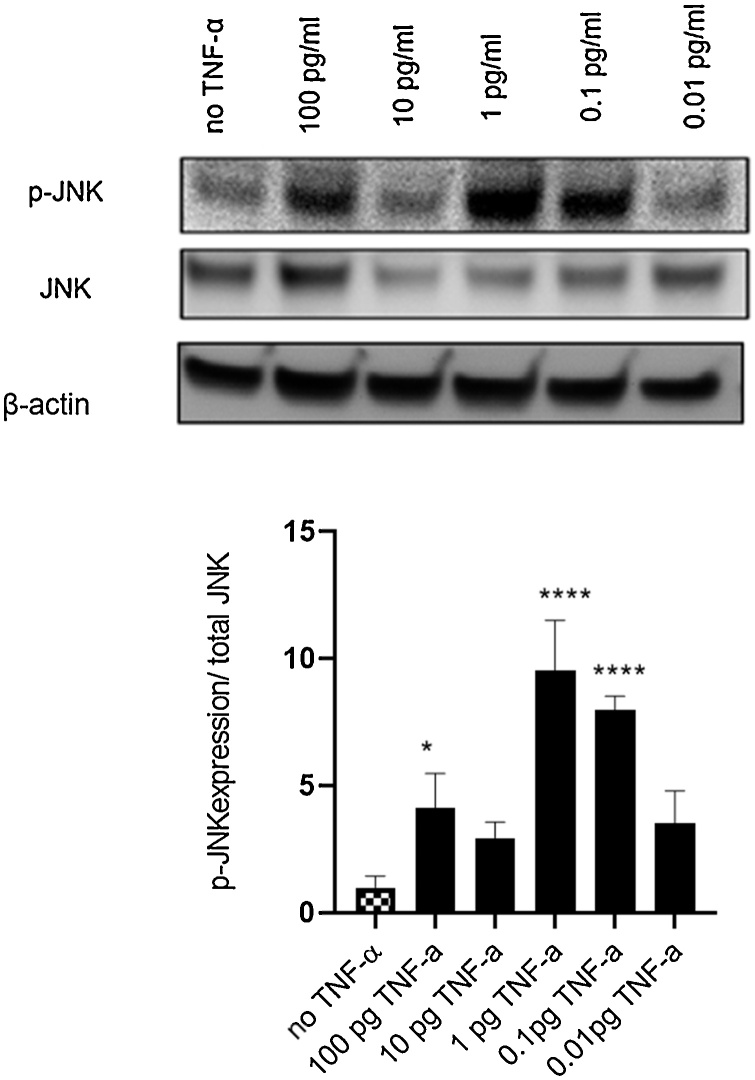
**Effect of TNF-α on JNK activation in colon cancer cells HCT116.** Levels of phosphorylated JNK are increased upon TNF-α treatment. Cells were treated with TNF-α dose range of (0.01-100 pg/mL) for 24 h. JNK and phosphorylated JNK (p-JNK) protein expression were determined by immunoblotting and quantified using Image J. Beta-actin was a loading control. Significance was assessed using one-way ANOVA (GraphPad Prism 8, (*p < 0.05, **p < 0.01, ***p < 0.001, **** *p* < 0.0001) compared to control. Data are presented as the ratio of p-IkB/total IkB with SEM from three independent cultures(n=3).

The response to TNF-α treatment was different for phosphorylation of JNK, with a bell-shaped dose-response that was maximal at 0.1–1.0 pg/ml (*p* <0.0001) (Fig. 6), indicating that TNF-α was activating the JNK pathway at physiological concentrations.

**Fig. 7:**
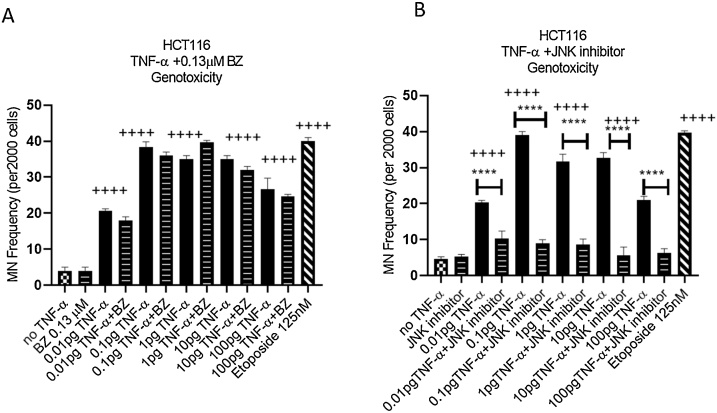
**JNK and NF-kB involvement in TNF-α induced DNA damage.** (**A**) genotoxicity of HCT116, cells were treated with 0.13μM of Bortezomib (BZ) with TNF-α dose range of (0.01-100 pg/mL) for 24 h. (B) genotoxicity of HCT116, cells were treated with 30μM of JNK inhibitor (SP600125) with TNF-α dose range of (0.01-100 pg/mL) for 24 h. Statistically Significant differences are shown for comparisons between inhibitor treated cells Vs. untreated cells. Significance was assessed using one-way ANOVA (GraphPad Prism 8, (**** *p* < 0.0001) and (++++ *p* < 0.0001) compared to control. Data are presented as a mean and error bars represent the SEM for independent cultures(n=3).

To further evaluate the involvement of NF-kB and JNK pathways in TNF-α genotoxicity, we inhibited these pathways in wild type HCT116 cells using bortezomib (0.13 μM) and SP600125 (30 μM) respectively, and MN formation were measured. Bortezomib was previously demonstrated to inhibit TNF-α-induced NF-kB signaling pathway (Westbrook et al., 2012). It is a strong proteasome inhibitor leading to IkB kinase (IKK) degradation, the enzyme which is responsible for IkB phosphorylation. In HCT116 cells bortezomib itself failed to elicit an induced MN frequency above that of the negative control. No changes were observed in the frequency of MN formation in the combination experiments with bortezomib and TNF-α-treated cells compared with cells treated with TNF-α alone (Fig. 7A), indicating that NF-kB pathway was unlikely to be involved in TNF-α-induced DNA damage despite being activated by the TNF-α treatment at the highest dose employed (100 pg/mL).

SP600125 is an ATP competitive inhibitor with specificity for JNK (Bennett et al., 2001). In cells treated with the JNK inhibitor alone, there was no change in the frequency of MN formation compared to the negative control indicating that the inhibitor was not genotoxic. However, in combination experiments of SP600125 and TNF-α, the frequency of MN formation was significantly reduced compared to TNF-α treatment alone across the entire TNF-α dose range (Fig. 7B), which suggested that the JNK pathway was involved in TNF-α-induced DNA damage.

## 4 Discussion

Inflammation and TNF-α expression is associated with many different tumor types including colorectal cancer (Charalambous et al., 2003, 2009; Stanilov et al., 2014). It is also known that inflammation and inflammatory mediators can affect the metabolic activation of chemical carcinogens and their ability to damage DNA (Malik et al., 2018); whether TNF-α can influence the DNA damaging effects of chemical carcinogens in colorectal cells is not clear. Therefore, the current study focused on the impact of TNF-α on regulation of DNA damage induced by BaP and PhIP, two dietary carcinogens present in cooked meats. While TNF-α has been previously shown to increase BaP-induced DNA damage in alveolar epithelial type II cells (Umannová et al., 2008; Lenka, 2013), a specific mechanism was not presented. To our best knowledge, we for the first time show that TNF-α itself induced DNA damage in human colonic HTC-116 cells, and co-treatment of cells with physiological concentrations of TNF-α with BaP- or PhIP exacerbated DNA damage. Interestingly, this TNF-α induced DNA damage did not show a clear dose-dependency and indeed was maximal at the 0.1–1.0 pg/ml levels. Inflammation mediators, including TNF-α, and hormones have been reported to show non-linear dose-responses (Corkey et al., 1991; Ip et al., 1992; Utsunomiya et al., 1996; Torrente et al., 2003; Kaneva et al., 2012). The reasons for these non-linear dose responses are not easy to explain, but optimal effective concentrations invariably peak at physiologically relevant levels, often in the pg/mL range, emphasizing the extreme potency of these physiologically active molecules. The increased levels of DNA damage in the TNF-α with BaP/PhIP combinations appeared to additive and not synergistic, suggesting independent mechanisms were operating for the chemical carcinogens versus the TNF-α.

To investigate the potential mechanisms involved, we first evaluated CYP1A1 and CYP1B1 expression, which are major CYP450 enzymes expressed in colonic tissue (Patel et al., 2014) that are involved in the activation of BaP and PhIP to their DNA damaging metabolites. Both CYP1A1 and CYP1B1 are AhR regulated genes. Several studies have reported that CYP1B1 expression was upregulated under conditions of inflammation (Kurzawski et al., 2012; Malaplate-Armand et al., 2003; Patel et al., 2014). Additionally, inflammation cytokines such as IL6 have been shown to upregulate CYP1B1 in colorectal cancer cells and mammary cells (Patel and Gooderham, 2015a, 2015b, Malik et al., 2018, 2019). TNF-α was reported to have an inhibitory effect on CYP1A1 expression in WB-F344 rat liver cells, however, it significantly increased expression of CYP1B1 by a mechanism that was independent of NF-κB activation (Umannová et al., 2007). It is pertinent to note that Umannova et al., used concentrations of TNF-α that were 10^3^ times greater than the physiological concentrations used in our experiments, making direct comparisons difficult. In our study, TNF-α alone did not induce alterations in CYP1A1 and CYP1B1 expression, nor in combined treatment with BaP or PhIP. This suggests that altered expression of CYP1A1 and CYP1B1 was not involved in TNF-a mediated genotoxicity in HCT116 cells.

Oxidative stress is known to induce MN frequency in target cells. Indeed, Yan et al., (Yan et al., 2006) reported that TNF-α (50 ng/mL) treatment of HCT116 cells induced oxidative stress and an associated increase in MN frequency, unfortunately this was a single supra-physiological dose study. We therefore examined whether physiological/pathophysiological concentrations of TNF−α induced ROS in HCT116 cells, but over the dose range 0.01–100 pg/mL no increase in ROS was detected. In interpreting these experiments, it is important to note that the concentration of TNF-a used by Yan et al., was 10^3^ higher than that used in our study and we therefore conclude that at physiological/pathophysiological levels of TNF-α, oxidative stress was not involved in the induction of the MN frequency.

The p53 status of cells is important in controlling a cellular response to DNA damage. In the case of BaP and PhIP induced DNA damage (herein assessed as micronuclei formation), the p53 status of the cell had little effect on the frequency of micronuclei formation. However, we found that p53 null cells were much more susceptible to TNF-α induced frequency of MN formation (∼2 fold), suggesting that p53 competent cells were able to attenuate TNF-α induced DNA damage. A caveat with these cell lines is the potential for the change in p53 status to alter multiple aspects of their cell biology and dynamics and comparisons should be made with caution. From our experiments it is not clear what the mechanisms involved are, but we speculate that TNF-α triggers a p53 DNA damage response that is dose-dependent. In the absence of p53 or at supra-physiological concentrations of TNF-a where the ability of p53 to respond is overwhelmed, additional biological events such as activation of apoptotic programmes and oxidative stress become increasingly important.

AhR and NF-κB physically interact and can modulate each other’s activity. AhR associates with the p65 (ReIA) subunit of NF-κB and as a result, activation of one pathway can down regulate the other (Tian et al., 1999; Ke et al., 2001). Here we report that treatment of cells with TNF-α increased NF-kB activation, as measured by phosphorylation of IKb. Consistent with this finding, a previous study in mice demonstrated the involvement of NF-κB signaling pathway in TNF-α genotoxicity as measured in the comet assay (Westbrook et al., 2012). However, in the present study, inclusion of the NF-kB pathway inhibitor bortezomib in incubations of cells with TNF-α failed to inhibit TNF-α mediated genotoxicity (micronucleus formation), indicating that TNF-α induced genotoxicity was not dependent on the NF-kB pathway. These studies suggest that different biochemistry is involved in TNF-α mediated DNA damage, *viz* transient strand breaks as measured by the comet assay versus clastogenic and aneugenic events as measured in the MN assay. The increased susceptibility of p53 null cells to TNF-α induced genotoxicity would support this concept whereby labile genome sites such as those picked up in a comet assay are readily repaired by a p53 proficient mechanism.

Activation of the JNK pathway is another cellular response frequently observed with TNF-α in multiple cell types (Wajant et al., 2003) and therefore, we investigated the impact of TNF-α on JNK activation. Here, we found that TNF-α-induced DNA damage was not only p53-dependent but also involved activation of JNK pathway since inhibition of JNK pathway using SP600125 significantly reduced the induced DNA damage. JNK is a distinct signaling pathway known for its role in the induction of cell apoptosis and is directly involved in the mitochondrial pathway of apoptosis (Dhanasekaran and Reddy, 2008). DNA damage is one of the pro-apoptotic mechanisms leading to cell death (Tournier, 2000). Activation of JNK by TNF-α is known to release cytochrome C from mitochondria into the cytoplasm, leading to caspase activation including caspase 3 which is involved in chromatin condensation and DNA fragmentation (Porter and Ja, 1999; Dhanasekaran and Reddy, 2008). However, the role of JNK in TNF-α mediated apoptosis requires further clarification (Wajant et al., 2003). How TNF-α mediated JNK pathway activation is involved in the increased MN frequency reported here is not clear from the current study and it will be important to explore further the mechanisms linking JNK pathway activation with DNA damage.

Collectively, these data suggest that TNF-α treatment of colonic HCT116 cells results in DNA damage that is mediated through the JNK pathway and likely to promote apoptotic pathways in a dose-dependent manner. Thus, treatment of cells with physiological doses of TNF-α (0.01 to 0.1 pg/mL) optimally induces micronuclei formation. When low dose TNF-α is combined with chemical carcinogens such as BaP and PhIP, induction of DNA damage is enhanced due to additive independent mechanisms most likely *via* JNK-mediated and CYP-mediated events respectively. These findings aid our understanding of the potential of inflammation mediators to contribute to chemical carcinogen activation, DNA damage and the process of carcinogenesis.

## 5 Conclusion

The present study has explored the effects of TNF-α alone and in combination with the food-derived chemical carcinogens benzo(a) pyrene (BaP) and 2-amino-1-methyl-6-phenylimidazo [4,5-b] pyridine (PhIP) on DNA damage induced in in CRC cells. On their own, each of these agents can induce DNA damage. Treatment with TNF-α along with the presence of BaP and PhIP exacerbated the induced DNA damage, compared to their effects in isolation. Whilst this was consistent with the notion that inflammation modulators contribute to DNA damage, our original hypothesis that TNF-α modulates BaP and PhIP induced DNA damage in colon cancer epithelial cells was not supported. Our data suggest that DNA damage induced by TNF-α versus BaP and PhIP operates through independent mechanisms, leading to additive and not synergistic levels of DNA damage. Our data suggests that the mechanisms involved are different with TNF-α causing activation of JNK signaling pathway leading to DNA damage that was sensitive to p53 status and likely involved pre-apoptotic events, whereas the chemical carcinogens are metabolically activated by CYP enzymes to their DNA damaging metabolites. These additive independent interactions are important in environments of ongoing inflammation that are metabolically competent and exposed to environmental procarcinogens and offer mechanistic support for the role of inflammation in carcinogenesis.

## Declaration of Competing Interest

The authors report no declarations of interest.
